# Perinatal outcomes of women with Müllerian anomalies

**DOI:** 10.1007/s00404-022-06557-6

**Published:** 2022-04-15

**Authors:** Si Wang, Kana Wang, Qing Hu, Hua Liao, Xiaodong Wang, Haiyan Yu

**Affiliations:** 1 Department of Obstetrics and Gynecology, West China Second University Hospital, Sichuan University, No. 20, 3rd section, South Renmin Road, Chengdu, 610041 Sichuan China; 2 Key Laboratory of Birth Defects and Related Diseases of Women and Children (Sichuan University), Ministry of Education, Chengdu, China

**Keywords:** Müllerian anomalies, Perinatal outcomes, Singleton pregnancy, Perinatal complications, Preterm delivery, Malpresentation

## Abstract

**Purpose:**

To investigate the perinatal outcomes of singleton pregnant women with Müllerian anomalies (MuAs).

**Methods:**

A retrospective cohort study was conducted on singleton pregnant women with MuAs who delivered at the West China Second University Hospital between January 1, 2009 and December 31, 2020.

**Results:**

Four hundred fifty-seven cases of MuAs were identified, with an incidence of 0.40%. The most common anomaly was a septate uterus (38.7%). Compared to the control group, the MuAs group had significantly higher incidences of perinatal complications, including preterm deliveries (PTDs) (27.4 vs. 9.8%, *P* < 0.001), preterm premature rupture of membranes (PPROM) (29.1 vs. 22.5%, *P* = 0.001), malpresentation (34.4 vs. 5.6%, *P* < 0.001), abruptio placentae (4.6 vs. 1.2%, *P* < 0.001), placental accreta/increta (19.7 vs. 11.8%, *P* < 0.001), and uterine rupture (2.8 vs. 1.6%, *P* = 0.035). The rates of in vitro fertilization and embryo transfer (IVF–ET), foetal growth restriction (FGR), and low birth weight were also significantly higher in the MuAs group (8.3 vs. 4.5%, *P* < 0.001; 2.6 vs. 0.9%, *P* = 0.001; 3.1 vs. 1.7%, *P* = 0.033, respectively). In the MuAs group, the incidence of PPROM was high in cases with unicornuate uterus (31.5%), and malpresentation was as high as 42.4 and 37.0% in cases with septate and didelphys uteri, respectively.

**Conclusion:**

The data suggest that pregnancy with MuAs may increase adverse perinatal outcomes, which calls for intensive supervision during pregnancy and delivery to reduce maternal and foetal complications. Individualized considerations should be emphasized according to the different categories of MuAs in pregnancies.

## Introduction

Female genital tract anomalies are congenital malformations of the female reproductive organs that result from the abnormal development of the Müllerian ducts (hence the phrase “Müllerian anomalies” [MuAs]) during embryonic formation and differentiation due to a variety of factors [1, 2] and involve the uterus, cervix, and vagina. Although women with MuAs are at an increased risk of infertility, miscarriage, and recurrent miscarriages [3], in recent years, there has been an increase in the success rate of pregnancy in patients with MuAs owing to the development of surgical correction and the use of assisted reproductive technologies [4–7]. Hence, the perinatal outcomes of pregnancy in these patients should be carefully monitored. Some investigators have shown that MuAs are associated with various adverse pregnancy outcomes, including PTDs, FGR, malpresentation, preterm premature rupture of membranes (PPROM), and hypertensive disorder complicating pregnancy (HDCP); however, their findings were not entirely consistent, and a majority of studies contained a relatively small sample size, with only a few providing a specific classification of the impact of different Müllerian anomalies on pregnancy outcomes [8–13]. In the present study, we retrospectively analysed the clinical data of pregnant women with MuAs who delivered at our hospital between January 2009 and December 2020 to provide a basis for clinical management strategies and improvements in perinatal outcomes.

## Materials and methods

We conducted a retrospective cohort study of women with singleton pregnancies who delivered between January 1, 2009 and December 31, 2020, at the West China Second University Hospital, a tertiary hospital in western China. This study was approved by the ethics committee of the West China Second University Hospital. The medical records of pregnant women were reviewed by the authors.

Inclusion and exclusion criteria: single live pregnant women diagnosed with MuAs before or after delivery were included in the study group. On the other hand, pregnant women with MuAs having multiple gestations, abortions, dead foetuses, or gestational age of less than 28 weeks upon delivery and those who underwent surgical correction before pregnancy were excluded from this study. The clinical data of pregnant women with non-MuAs who were admitted to our hospital during the same period for singleton pregnancies and live births were included in the control group.

MuAs were diagnosed according to the criteria of the European Society of Human Reproduction and Embryology (ESHRE), classification system of the European Society of Gynaecological Endoscopy (ESGE), and Chinese expert consensus [14–17].

Patients with MuAs were included in the study group, and patients without MuAs were included in the control group.

Maternal data collected included demographic characteristics, obstetric history, classifications of MuAs, delivery age, complications, and mode of delivery. In addition, neonatal data included gestational age at delivery and birth weight.

Statistical analyses were performed using SPSS version 22.0. Quantitative variables were compared using Student’s *t* test, and categorical variables were assessed using the chi-squared test, with continuity correction and Fisher's exact test where appropriate. Statistical significance was set at* P* < 0.05.

## Results

During the study period, 114,020 women with singleton pregnancies delivered at our institution, including 113,563 cases with non-MuAs (control group) and 457 cases with MuAs (study group). Therefore, the incidence rate of singleton pregnancies with MuAs was 0.40%.

The maternal age of the 457 patients with MuAs, including 355 nulliparous women (77.7%) and 102 multigravida women (22.3%), was 29.82 ± 4.08 years (19–49 years). In the study group, 243 male (53.2%) and 214 female (46.8%) babies were delivered. The prenatal diagnosis rate for MuAs was 74.4% (340/457), as determined using ultrasonography, hysteroscopy, laparoscopy, hysterosalpingography, and magnetic resonance imaging, while the remaining 117 cases were diagnosed at delivery.

We depicted three categories of pregnancies with MuAs: uterine anomalies, vaginal anomalies, and uterine anomalies combined with vaginal anomalies. The most common type of anomaly in the study group was septate uterus (38.7%), while the other MuAs that we observed were unicornuate (27.8%), arcuate (10.9%), didelphys (5.9%), bicornuate uterus (4.2%), transverse vaginal septum (6.3%), longitudinal vaginal septum (0.7%), and uterine anomalies combined with vaginal anomalies (complex malformations, 5.5%). The detailed information is presented in (Table 1).

**Table 1:** Types of Müllerian anomalies

Categories	Cases	%
Septate	177	38.7
Unicornuate	127	27.8
Arcuate	50	10.9
Didelphys	27	5.9
Bicornuate	19	4.2
Transverse vaginal septum	29	6.3
Longitudinal vaginal septum	3	0.7
Complex malformation	25	5.5
Total	457	100

Women in the MuAs group had a significantly higher incidence of PTDs than the control group (27.4 vs. 9.8%, *P* < 0.001), and the risk for PTDs at < 37, < 34, and < 32 weeks of gestation was significantly higher in the MuAs group. For the cases with MuAs, the risk ratios for the occurrence of PTDs at < 37 weeks, < 34 weeks, and < 32 weeks are detailed in (Table 2).

**Table 2:** Risk ratio for preterm delivery

Characteristics	MuAs (*n* = 457)	No MuAs (*n* = 113,563)	*P* value	OR (95% CI)
Preterm (wks), *n* (%)	125 (27.4%)	11,152 (9.8%)	< 0.001	3.458 (2.81–4.25)
< 37	71 (15.5%)	7549 (6.6%)	< 0.001	2.583 (2.00–3.33)
< 34	29 (6.3%)	1435 (1.3%)	< 0.001	5.294 (3.62–7.74)
< 32	25 (5.4%)	1396 (1.2%)	< 0.001	4.650 (3.10–6.98)

*MuAs* Müllerian anomalies, *wks* weeks

The incidence of PTDs, PPROM, malpresentation, placental abruption, placenta accreta/increta, FGR, uterine rupture (complete/incomplete), and caesarean section were significantly higher in the study group than in the control group (*P* < 0.05). The birth weight of the new-borns in the study group was 2924.06 ± 618.74 g, which was significantly lower than that of new-borns in the control group (3233.03 ± 477.25 g) (*P* < 0.001). There were no significant differences in the incidence of placenta previa, oligohydramnios, HDCP, postpartum haemorrhage (PPH), stained amniotic fluid, or hysterectomy rate between the two groups (*P* > 0.05). A comparison of perinatal outcomes between the two groups is shown in (Table 3).

**Table 3:** Perinatal outcomes of women with and without MuAs

Characteristics	MuAs (*n* = 457)	No MuAs (*n* = 113,563)	*P* value	OR (95% CI)
Preterm	125 (27.4%)	11,152 (9.8%)	< 0.001	3.458 (2.81–4.25)
PPROM	133 (29.1%)	25,503 (22.5%)	0.001	1.41 (1.16–1.74)
Malpresentation	157 (34.4%)	6353 (5.6%)	< 0.001	8.832 (7.27–10.73)
Placenta previa	26 (5.7%)	7725 (6.8%)	0.355	0.826 (0.56–1.23)
Abruptio placentae	21 (4.6%)	1390 (1.2%)	< 0.001	3.887 (2.50–6.04)
Oligohydramnios	7 (1.5%)	3252 (2.7%)	0.088	0.528 (0.25–1.11)
HDCP	13 (2.8%)	5385 (4.7%)	0.060	0.588 (0.34–1.02)
Placenta accreta/increta	90 (19.7%)	13,401 (11.8%)	< 0.001	1.833 (1.45–2.31)
Uterine rupture	13 (2.8%)	1766 (1.6%)	0.035	1.854 (1.07–3.22)
PPH	17 (3.7%)	4583 (4.0%)	0.812	0.919 (0.57–1.49)
Stained amniotic fluid	29 (6.3%)	5811 (5.1%)	0.241	1.256 (0.86–1.83)
^a^Hysterectomy	1 (0.2%)	265 (0.2%)	1.000	0.938 (0.13–6.70)
IVF–ET	38 (8.3%)	5097 (4.5%)	< 0.001	1.930 (1.38–2.69)
FGR	12 (2.6%)	1022 (0.9%)	0.001	2.969 (1.67–5.29)
Low birthweight	14 (3.1%)	1957 (1.7%)	0.033	1.802 (1.06–3.07)
Macrosomia	1 (0.2%)	4316 (3.8%)	< 0.001	0.056 (0.01–0.40)

*MuAs* Müllerian anomalies, *PROM* premature rupture of membranes, *PPROM* preterm premature rupture of membranes, *IVF–ET* in vitro fertilization and embryo transfer, *FGR* foetal growth restriction, *HDCP* hypertensive disorder complicating pregnancy, *PPH* postpartum haemorrhage

^a^The Chi-squared test with continuity correction

In the study group of pregnant women with different types of MuAs, complex malformations had the highest rate of PTDs (40.0%) and PPROM (48%), and the highest incidence of malpresentation was 42.4% in the septate uterus. The incidence of abruptio placentae (12%) was highest for patients with arcuate uterus; the incidences of FGR (10.5%), placenta accreta/increta (26.3%), and low birth weight foetuses (5.3%) were highest for patients with bicornuate uterus; and the incidences of uterine rupture (4.7%) and IVF–ET (12.6%) were highest for patients with unicornuate uterus. The perinatal outcomes of pregnant women with different types of Müllerian anomalies are shown in (Table 4).

**Table 4:** Perinatal outcomes of pregnant women with different types of Müllerian anomalies

Characteristics	Septate (*n* = 177)	Unicornuate (*n* = 127)	Arcuate (*n* = 50)	Didelphys (*n* = 27)	Bicornuate (*n* = 19)	Transverse vaginal septum (*n* = 29)	Longitudinal vaginal septum (*n* = 3)	Complex anomalies (*n* = 25)
Preterm	52 (29.4%)	35 (27.6%)	12 (24.0%)	8 (29.6%)	2 (10.5%)	6 (20.7%)	0	10 (40%)
PPROM	50 (28.2%)	40 (31.5%)	13 (26.0%)	6 (22.2%)	5 (26.3%)	7 (24.1%)	0	12 (48%)
Malpresentation	75 (42.4%)	40 (31.5%)	15 (30.0%)	10 (37.0%)	5 (26.3%)	3 (10.3%)	0	9 (36%)
Placenta previa	6	11	2	3	1	2	0	1
Abruptio placentae	12 (6.8%)	1 (0.8%)	6 (12%)	1 (3.7%)	0	0	0	1 (4%)
Oligohydramnios	5	1	0	1	0	0	0	0
HDCP	4	5	0	3	0	0	0	1
Placental accreta/increta	43 (24.3%)	20 (15.7%)	10 (20%)	6 (22.2%)	5 (26.3%)	2 (6.9%)	0	4 (16.0%)
Uterine rupture	5 (2.8%)	6 (4.7%)	1 (2%)	1 (3.7%)	0	0	0	0
PPH	6	5	1	1	0	2	0	2
Stained amniotic fluid	6	15	3	2	1	1	0	1
Hysterectomy	0	0	0	0	0	0	0	1
IVF–ET	15 (8.5%)	16 (12.6%)	3 (6%)	1 (3.7%)	1 (5.3%)	1 (3.4%)	0	1 (4%)
FGR	5 (2.8%)	3 (2.4%)	1 (2%)	1 (3.7%)	2 (10.5%)	0	0	0
Low birthweight	6 (3.4%)	6 (4.7%)	0	1 (3.7%)	1 (5.3%)	0	0	0
Macrosomia	1 (0.6%)	0	0	0	0	0	0	0

*PROM* premature rupture of membranes, *PPROM* preterm premature rupture of membranes, *IVF–ET* in vitro fertilization and embryo transfer, *FGR* foetal growth restriction, *HDCP* hypertensive disorder complicating pregnancy, *PPH* postpartum haemorrhage

In the study group, out of 457 patients with MuAs, 104 had vaginal deliveries (95 with eutocia, six with assisted breech delivery, two with vacuum-assisted vaginal delivery, and one with forceps delivery), and 353 had caesarean sections (77.2%). The caesarean delivery rate was 79.5% (318/400) for uterine anomalies, 76% (19/25) for complicated anomalies, and 50.0% (16/32) for vaginal anomalies; the highest caesarean delivery rate was 90% for patients with arcuate uterus. The rate of caesarean section in the study group was significantly higher than that in the control group (77.2 vs. 61.8%, *P* < 0.001), and the risk ratio for caesarean section with MuAs showed an OR = 2.100, with a 95% CI = 1.69–2.61. The perinatal outcomes and modes of delivery of pregnant women with different types of Müllerian anomalies are shown in (Table 5 and Table 6).

**Table 5:** Mode of delivery for women with and without MuAs

Mode of delivery	MuAs (*n* = 457)	No MuAs (*n* = 113,563)	*P* value
Caesarean section	353 (77.2%)	70,159 (61.8%)	*P* < 0.001
Vaginal delivery	104 (22.8%)	43,404 (38.2%)	

*MuAs* Müllerian anomalies

**Table 6:** Mode of delivery in pregnant women with different types of Müllerian anomalies

Characteristics	Septate (*n* = 177)	Unicornuate (*n* = 127)	Arcuate (*n* = 50)	Didelphys (*n* = 27)	Bicornuate (*n* = 19)	Transverse vaginal septum (*n* = 29)	Longitudinal vaginal septum (*n* = 3)	Complex anomalies (*n* = 25)
Caesarean	126 (71.2%)	107 (84.5%)	45 (90.0%)	22 (81.5%)	16 (84.2%)	16 (55.2%)	2 (66.7%)	19 (76.0%)
Vaginal	47 (26.6%)	19 (15.0%)	5 (10.0%)	5 (18.5%)	3 (15.8%)	11 (37.9%)	0	5 (20.0%)
Assisted	4 (2.2%)	1 (0.8%)	0	0	0	2 (6.9%)	1 (33.3%)	1 (4.0%)

A scarred uterus was the primary indication for caesarean sections (27.1%, 19,013/70159) in the control group, and a breech baby was the primary indication for caesarean section in the MuAs group (33.4%, 118/353). In the study group, caesarean section due to MuAs was requested by only 14.7% (52/353) of patients.

Exploration of the history of pregnancy in patients with MuAs showed that there were 805 pregnancies (including spontaneous abortions and live births, excluding artificial abortion). The rate of spontaneous abortions was 26.6% (214/805), which was far higher than the rates of 7.45% reported in other studies in China [18] and 14.6% reported in other countries [19] (i.e., 26.6 vs. 7.45%, *P* < 0.001; 26.6 vs. 14.6%, *P* < 0.001).

## Discussion

Female genital tract anomalies often result from the abnormal development of Müllerian or paramedian ducts and may be associated with genetic mutations or environmental factors [20]. Previous studies have shown that the proportion of pregnancies with Müllerian anomalies was 0.48–0.49% [11, 21]. In this study, the percentage of Müllerian anomalies associated with pregnancy was 0.40%, which is similar to the conclusion of previous studies. (The difference in the proportion of pregnancies in previous studies was due to the gestational age at prematurity being 24 to 37 weeks; however, the gestational age at prematurity was 28–37 weeks in our study, so there was a slight difference.)

There is a clinically diverse presentation of female MuAs and a certain number of missed diagnoses in clinical practice. In this study, the prenatal diagnosis rate was only 74.4%; however, the diagnosis of MuAs before or early in pregnancy is important for enhancing perinatal management and reducing maternal and infant complications. Therefore, corresponding clinical management is required during premarital screening, preconception screening, and early pregnancy. This includes a detailed history and physical examination, especially for patients with a family history of MuAs, dysmenorrhoea, infertility, habitual miscarriage, malpresentation, or dystocia, which are performed to determine the development of the vulva, vagina, cervix, and uterus. If needed, the type of MuAs should be further clarified by combining appropriate additional examinations, such as hysteroscopy, hysterosalpingography, MRI, and three-dimensional (3D) ultrasonography [22–24]. 3D image reconstruction and 3D-printed models based on thin MRI (pelvic MRI performed with a thin layer scan of 1 mm) data have developed into novel techniques for the non-invasive preoperative diagnosis of complex reproductive tract anomalies [25, 26]. With proper clinical management, the early detection of MuAs can further improve the diagnostic rate before and during early pregnancy.

The data from this study revealed that the spontaneous abortion rate in patients with MuAs was 26.6%, which was significantly higher than the general abortion rate. El Hachem reported that more than 19% of patients with uterine abnormalities had recurrent miscarriages [27], and a study by Saravelos also showed that approximately 17% of recurrent-abortion patients exhibited MuAs [28]. Thus, our findings are in agreement with previously published data. The reason for this high proportion may be related to poor blood supply to the myometrium due to abnormal development of the vagina, cervix, and/or uterus, irregular shape of the uterine cavity, small uterine cavity volume, endometrial dysplasia, or abnormal vascular distribution in patients with reproductive tract malformation after pregnancy, which may affect the implantation of zygotes, placental formation, and/or embryonic development [29, 30].

The results of this study showed that PTDs, PPROM, malpresentation, placental abruption, placenta accreta/increta, and other pregnancy complications increased during pregnancy in women with MuAs and that FGR was also significantly higher in the MuAs group than in the control group. The rate of caesarean section was significantly higher in the group with MuAs, and the birth weight of the babies was significantly lower than that of the control group. Some investigators reported that the incidence of PPROM, pathologic presentation, placental abruption, and oligohydramnios after pregnancy in patients with MuAs was significantly elevated and that the rate of caesarean section in patients was dramatically increased [8–10]. Cahen-Peretz et al. [11] also posited that Müllerian anomalies constitute an independent risk factor for premature delivery, placental abruption, caesarean section, FGR, and foetal position abnormalities. A meta-analysis by Venetis et al. [31] uncovered a correlation between uterine growth abnormalities and an increased incidence of perinatal complications such as preterm delivery, abnormal foetal position, FGR, and placental abruption [32, 33]. This may be attributed to the reduced cervical connective tissue and increased muscle composition that are insufficient to withstand the increased and asymmetrical uterine pressure after pregnancy; hence, preterm delivery, abnormal foetal positioning, and other risks were significantly increased.

The incidences of placenta previa and postpartum haemorrhage in the group with MuAs showed no significant differences, which was consistent with the results of Cahen-Peretz et al. [21] and Shim et al. [34]. Although Fox et al. [8] found that reproductive tract malformations increased the incidence of hypertensive diseases during pregnancy, we did not find any statistical significance compared with the control group. Although the sample size of our investigation was large, it contained data from only one centre. Thus, the relationship between MuAs associated with pregnancy and HDCP, placenta previa, and postpartum haemorrhage still needs to be analysed further using larger sample sizes and the involvement of additional centres.

Our study results showed that malpresentation, PPROM, PTDs, and placental accreta/increta in patients with MuAs were significantly higher than those in the control group. Therefore, we need to be more attentive to the maternal and foetal conditions during pregnancy and delivery to prevent complications. We demonstrated that although only 14.7% of patients underwent caesarean section because of MuAs, malpresentation, uterine scarring, and other obstetric complications were still the principal indications for caesarean section in patients with MuAs. Although pregnancy for women with MuAs was not an absolute indication for caesarean section, MuAs could lead to an increased incidence of dystocia and a particularly elevated incidence of foetal position abnormality; with foetal transfer not appropriate, the mode of delivery should be selected according to individual differences in clinical practice. However, the caesarean delivery rate in the control group in our study was 61.8%, which is higher than that reported in previous studies [10, 11]. This may be due to the special national conditions that our hospital, as a centre for maternal critical care in the southwest, namely, that it receives a large number of difficult and critically ill patients referred from outside hospitals.

Women with MuAs detected before pregnancy and those with uterine malformations without clinical manifestations are generally not treated. Laparoscopic correction for uterine or vaginal malformations is considered to improve pregnancy and pregnancy outcomes in patients with infertility, repeated abortion, or other adverse pregnancy histories [35, 36]. If MuAs were found to be present during caesarean section or delivery (and considering that the blood supply to the uterus is rich during pregnancy and that diorthosis is not deemed appropriate), correction of the deformities could be performed concomitantly if necessary, depending on the intraoperative situation. There are no recognized recommendations or guidelines as to whether a hysterectomy should be performed during caesarean section in women with no history of adverse pregnancy. However, in the case of a unicornuate uterus combined with a rudimentary uterus, if the rudimentary uterus is combined with a functional endometrium that may cause periodic abdominal pain and if there is a risk of uterine rupture after pregnancy in the rudimentary uterus, hysterectomy of the rudimentary uterus and ipsilateral salpingectomy can be performed concurrently during the caesarean section [37].

The presence of Müllerian anomalies during pregnancy may increase perinatal maternal and infant complications and the incidence of adverse pregnancy outcomes. Thus, for patients with MuAs, clinicians need to extend early detection, diagnosis, and treatment. As Müllerian anomalies constitute a high-risk pregnancy, intensive supervision should be taken to reduce maternal and foetal complications: routine pre-pregnancy check-ups, ultrasound, hysteroscopy or hysteron-salpingography should be performed before pregnancy for women at high risk (dysmenorrhoea, habitual miscarriage, malposition or dystocia) to detect uterine malformations. If MuAs are found during pregnancy, the type of anomaly should be promptly identified, the impact on the mother and foetus should be evaluated, and the frequency of prenatal care should be increased appropriately. If PPROM or signs of preterm delivery occur, antibiotics and uterine contraction inhibitors should be used appropriately, and the gestational age should be prolonged as much as possible under close monitoring. However, there are no clear guidelines on the timing of delivery in pregnancies with Müllerian anomalies. The individual timing of delivery and mode of delivery should be determined according to the specific circumstances, with the aim of reducing maternal and child complications. Although MuAs are not an absolute indication for caesarean section, they should be performed for conditions such as malpresentation, FGR or a history of adverse pregnancy. In addition, we need to be more attentive to the retained placenta, postpartum haemorrhage and other complications after delivery to ensure the safety of mothers and neonates.

The strengths of this study are our comparison of the perinatal outcomes of a large sample of singleton pregnancies among women with MuAs and presentation of the perinatal outcomes of women with different types of MuAs. The limitations of this study are its retrospective nature and use of data from a single centre. Further studies should be conducted with the involvement of multiple centres.

## Data Availability

All data are fully available without restriction within the manuscript.
